# Clinical and Genomic Characterization of Secondary Rectal Cancer After Radiotherapy for Prostate Cancer

**DOI:** 10.1001/jamanetworkopen.2025.1039

**Published:** 2025-03-18

**Authors:** Dana M. Omer, Farheen Shah, Anisha Luthra, Chin-Tung Chen, Christina I. Lee, Hannah Williams, Henry Walch, Floris S. Verheij, Roni Rosen, Janet Alvarez, Canan Firat, Georgios Karagkounis, Martin R. Weiser, Maria Widmar, Iris H. Wei, Emmanouil P. Pappou, Garrett M. Nash, J. Joshua Smith, Walid K. Chatila, Paul B. Romesser, Jinru Shia, Philip B. Paty, Julio Garcia-Aguilar, Francisco Sanchez-Vega

**Affiliations:** 1 Department of Surgery, Colorectal Service, Memorial Sloan Kettering Cancer Center, New York, New York; 2 Department of Epidemiology and Biostatistics, Memorial Sloan Kettering Cancer Center, New York, New York; 3 Department of Pathology, Memorial Sloan Kettering Cancer Center, New York, New York; 4 Department of Radiation Oncology, Colorectal Service, Memorial Sloan Kettering Cancer Center, New York, New York

## Abstract

**Importance:**

Patients treated with radiotherapy (RT) for prostate cancer (PC) have increased risk of secondary rectal cancer (SRC) and more limited treatment options.

**Objective:**

To assess the tumor molecular profile, clinical characteristics, and oncologic outcomes of SRC after PC and compare them with those of primary rectal cancer (PRC).

**Design, Setting, and Participants:**

This case-control study included patients with SRC diagnosed 5 or more years after RT for PC and patients with PRC who were treated at Memorial Sloan Kettering Cancer Center in New York between February 1, 1994, and September 31, 2022.

**Main Outcomes and Measures:**

Clinical information and DNA sequencing data were analyzed. Oncologic outcomes were compared between patients with SRC and clinically matched patients with PRC using log-rank tests and Cox proportional hazards regression models. Numerical and categorical variables were compared using the Wilcoxon rank sum test and Fisher exact test, respectively.

**Results:**

The analysis included 604 male patients with PRC (71.6%; median age, 55 [IQR, 46-66] years) and 64 male patients with SRC (median age, 78 [IQR, 72-82] years). Patients with SRC had more distal rectum (37 of 63 [58.7%] vs 131 of 581 [22.5%]; *P* < .001) and anterior rectal wall (20 of 57 [35.1%] vs 67 of 496 [13.5%]; *P* < .001) tumors, were less likely to receive neoadjuvant treatment (33 of 64 [51.6%] vs 570 of 604 [94.4%]), and had shorter 5-year overall survival (45.7% vs 64.9%; *P* = .01) and disease-free survival (40.3% vs 71.2%; *P* = .006) compared with clinically matched patients with PRC. Targeted DNA sequencing data from 31 SRC tumors identified lower mutational burden (median, 4.4 [IQR, 3.2-6.7] per megabase [Mb] vs 5.8 [IQR, 4.4-7.0] per Mb; *P* = .047), lower frequency of *APC* alterations (15 [48.4%] vs 432 [79.9%]; *P* < .001), and higher rates of *SMAD4* inactivation (8 [25.8%] vs 54 [10.0%]; *P* = .01) compared with 541 PRC tumors. Whole-exome sequencing data from 17 SRC tumors identified a higher rate of frameshift deletions compared with 28 PRC tumors (median, 5.0 [IQR, 4.0-9.0] vs 2.5 [IQR, 1.0-4.2] variants; *P* < .001).

**Conclusions and Relevance:**

In this case-control study, patients with SRC after RT for PC had worse survival and different molecular profiles than patients with PRC. These findings may help improve the clinical management of SRC.

## Key Points

**Question:**

Are there clinical or genomic differences between patients who develop secondary rectal cancer (SRC) following prostate radiotherapy and patients with primary rectal cancer (PRC)?

**Findings:**

This case-control study of 64 patients with SRC found that these patients were significantly older, more often presented with tumors in the distal rectum and the anterior rectal wall, and had worse survival outcomes compared with patients with PRC. Patients with SRC also had significantly lower mutational burden, fewer *APC* alterations, and higher rates of frameshift deletions and *SMAD4* inactivation.

**Meaning:**

The findings suggest that distinct clinical and genomic features reflect differences in the underlying biologic characteristics of SRC.

## Teaser

This case-control study compares the tumor molecular profiles, clinical characteristics, and oncologic outcomes between patients with primary rectal cancer and patients who develop secondary rectal cancer after radiotherapy for prostate cancer.

## Introduction

Several pelvic malignant tumor types undergo radiotherapy (RT) as part of treatment. This may damage pelvic tissues and adjacent organs, potentially causing chronic discomfort and urinary tract, sexual, and gastrointestinal tract dysfunction.^1^ The genotoxic effect of RT is also associated with the development of secondary cancers, which are often diagnosed many years after initial exposure.^2,3,4,5,6,7^ To be considered a radiation-associated tumor, the secondary malignant tumor should originate in the field irradiated during the treatment of the initial tumor and develop at least 5 years after RT.^7,8^

Population studies have shown that patients with prostate cancer (PC) treated with RT have an increased risk of developing secondary rectal cancer (SRC) compared with patients with PC treated without RT and with the general population.^3,5,7,8,9,10^ In the US, the lifetime risk of developing PC is 11%,^11^ with approximately 300 000 new diagnoses annually, and around 3.4 million men are currently living with this disease.^12^ As 1 in 4 patients with PC receive external beam radiotherapy (EBRT), brachytherapy (BRT), or both as part of their treatment,^13^ the number of patients exposed to the risk of SRC is potentially significant.

Tumors secondary to RT often carry distinct molecular alterations and may have different biological behavior compared with primary tumors originating in the same organ.^5,14,15,16,17^ Patients with SRC after RT for PC have been reported to have shorter survival compared with matched treatment-naive patients,^5^ but it is unclear whether this difference in survival is due to differences in patient characteristics, available treatment options, or tumor biologic characteristics.^10^ To address this gap in knowledge, we compared the tumor molecular profile, clinical characteristics, and oncologic outcomes in patients with SRC developing after RT for PC with those in patients with primary rectal cancer (PRC).

## Methods

### Patient Selection

This case-control study was approved by the institutional review board at Memorial Sloan Kettering Cancer Center (MSKCC) in New York, and informed consent was obtained from all patients. We identified patients with colorectal adenocarcinoma and a history of PC treated at MSKCC between February 1, 1994, and September 31, 2022. Patients with an adenocarcinoma located 12 cm or less from the anal verge that was diagnosed at least 5 years after completing RT for PC were considered to have SRC (eFigure 1 in Supplement 1). A control group of patients with rectal cancer without a history of PC or pelvic RT treated during the same period was used for comparison. Detailed clinical information for all patients is provided in eTable 10 in Supplement 2. Additional information for patients who underwent targeted DNA sequencing and whole-exome sequencing (WES) is provided in eTables 11 and 12 in Supplement 2 and eFigure 1 in Supplement 1. This study adhered to the reporting requirements of the Strengthening the Reporting of Observational Studies in Epidemiology (STROBE) statement.^18^

### Oncologic Outcomes

We compared overall survival (OS), disease-free survival (DFS), local recurrence–free survival, and distant recurrence–free survival (DRFS) between male patients with SRC and a clinically matched cohort of male patients with PRC. Local recurrence was defined as locoregional tumor reappearance after surgical resection of the rectal cancer. Distant metastasis was defined as a recurrence in distant organs and nonlocoregional lymph nodes. Time 0 was the date of rectal cancer diagnosis.

### Sample Sequencing and Tumor Profiling

Biopsy samples from patients with SRC or PRC were retrieved from formalin-fixed paraffin-embedded blocks. Thirty-one tumor specimens with sufficient material for molecular profiling were analyzed using the Memorial Sloan Kettering-Integrated Mutation Profiling of Actionable Cancer Targets (MSK-IMPACT) platform, a targeted DNA-sequencing panel that identifies somatic mutations, copy number changes, and fusions in a panel of 341 to 505 cancer-associated genes.^19^ Matched normal blood samples were used for filtering germline variants for 24 of the 31 patients (77.4%). Normal adjacent tissue samples (3 patients [9.7%]) or pooled normal DNA (4 patients [12.9%]) were used for the remaining patients. Seventeen of the 31 SRC samples (54.8%) had sufficient material for WES recapture. WES was done at ×150 for tumors and ×70 for matched normal specimens, including blood specimens for 14 patients and adjacent noncancer tissue specimens for 3 patients.

### Computational Genomics Analysis

Upstream processing of MSK-IMPACT data was performed as previously described.^20,21,22^ Tumor mutational burden (TMB) was defined as the number of nonsynonymous mutations divided by the number of million bases sequenced, and the fraction of genome altered (FGA) was defined as the fraction of the profiled genome exhibiting log_2_ copy number variation (gain or loss) greater than 0.2. The FACETS algorithm^23^ was used for allele-specific copy number analyses. Microsatellite instability status was established using MSIsensor and a threshold score of at least 10.^24^ The OncoKB knowledge base was used to distinguish oncogenic somatic alterations from variants of unknown significance.^25,26^ WES data were processed using the TEMPO pipeline developed at MSKCC.^27^ Mutational signatures were analyzed using the dictionary of COSMIC, version 3 signatures.^28^

### Statistical Analysis

Statistical analysis was performed using R, version 4.3.2 (R Project for Statistical Computing). Baseline characteristics between the group with SRC and the group with PRC were compared using the Wilcoxon rank sum test for continuous variables and the Fisher 2-sided exact test for categorical variables. To compare the oncologic outcomes of SRC and PRC, we used propensity score matching with a 1:1 ratio using the R package MatchIt^29^ and the nearest neighbor method. Patients were matched for sex, age at rectal cancer diagnosis, cancer stage, tumor distance from the anal verge, tumor size, and tumor circumferential location (eFigure 1 in Supplement 1). Survival was evaluated using Kaplan-Meier curves and the log-rank test. Multivariate survival analyses were performed using Cox proportional hazards regression models, with patients stratified according to their clinical stage. For the genomic comparisons, patients with SRC with MSK-IMPACT data were compared with the entire cohort with PRC with available MSK-IMPACT data and with a propensity score–matched cohort (1:2 ratio) (eFigure 1 in Supplement 1). Two-sided *P* values less than .05 were considered statistically significant. The Benjamini-Hochberg method was used to correct for multiple hypothesis testing, and *q* values less than .10 were considered significant. We considered that a mutational signature was detected in a given sample if at least 10% of its mutations could be attributed to it and the detection *P* value was less than .05. We compared the frequency of detection in SRC and PRC specimens for each signature using the Fisher exact test.

## Results

We initially identified 498 patients with colorectal adenocarcinoma and a history of PC; 64 patients had an adenocarcinoma located 12 cm or less from the anal verge that was diagnosed 5 or more years after completing RT for PC and were considered to have SRC (100% males; median age, 78 [IQR, 72-82] years) (Table 1). The control group comprised 843 patients (231 female [27.4%]; 612 male [72.6%]) with rectal cancer without a history of PC or pelvic RT. Among the 604 males with PRC, the median age was 55 (IQR, 46-66) years. In the control group, MSK-IMPACT data were available for 541 patients with PRC (223 females [41.2%]; 318 males [58.8%]) and WES data were available for 28 patients (eFigure 1 in Supplement 1).

**Table 1.** Comparison of Clinical Characteristics of Patients With SRC vs Those With PRC

Clinical characteristic	Patients, No./total No. (%)		*P* value
	PRC (n = 604)	SRC (n = 64)	
Age at rectal cancer diagnosis, median (IQR), y	55 (46-66)	78 (72-82)	<.001
cT classification			
1-2	63/555 (11.4)	16/52 (30.8)	<.001
3	419/555 (75.5)	26/52 (50.0)	
4	73/555 (13.1)	10/52 (19.2)	
Not reported	49	12	NA
cN classification			
Negative	109/574 (19.0)	32/54 (59.3)	<.001
Positive	465/574 (81.0)	22/54 (40.7)	
Not reported	30	10	NA
cTNM stage			
I	30/604 (5.0)	15/54 (27.8)	<.001
II	75/604 (12.4)	17/54 (31.5)	
III	370/604 (61.3)	16/54 (29.6)	
IV	129/604 (21.3)	6/54 (11.1)	
Not reported	0	10	NA
Tumor size			
Median (IQR), cm	4.60 (3.70-6.10)	3.50 (2.30-4.35)	<.001
<4 cm	176/554 (31.8)	32/55 (58.2)	<.001
4-8 cm	336/554 (60.6)	21/55 (38.2)	
>8 cm	42/554 (7.6)	2/55 (3.6)	
Not reported	50	9	NA
Tumor location in distal rectum			
No	450/581 (77.5)	26/63 (41.3)	<.001
Yes	131/581 (22.5)	37/63 (58.7)	
Not reported	23	1	NA
Circumferential tumor location			
Anterior	67/496 (13.6)	20/57 (35.1)	<.001
Circumferential	142/496 (28.6)	4/57 (7.0)	
Lateral	74/496 (14.9)	7/57 (12.3)	
Partially circumferential	153/496 (30.8)	10/57 (17.5)	
Posterior	60/496 (12.1)	16/57 (28.1)	
Not reported	108	7	NA

Abbreviations: NA, not applicable; PRC, primary rectal cancer; SRC, secondary rectal cancer.

### Clinical Characteristics of Patients With SRC Who Received RT for PC

In total, 64 patients were identified to have SRC. Radiotherapy for PC consisted of EBRT (31 [48.4%]), interstitial BRT (19 [29.7%]), or a combination of both (14 [21.9%]) (eFigure 1 in Supplement 1). The median dose of radiation received for the treatment of prostate cancer was 7560 (IQR, 5040-7560) cGy for patients treated with EBRT and 14 400 (IQR, 14 400-14 400) cGy for patients treated with BRT. The median latency period from prostate RT completion to diagnosis of rectal cancer was 11.50 (IQR, 7.00-17.25) years, and median follow-up from diagnosis of rectal cancer was 2.38 (IQR, 1.97-3.26) years.

### Comparison of Clinical Characteristics and Treatment Between the Groups With SRC and PRC

Compared with the reference set of 604 male patients with PRC, the 64 patients with SRC were older and more often diagnosed with stage I disease (15 of 54 [27.8%] vs 30 of 604 [5.0%]; *P* < .001) (Table 1 and eFigure 2 in Supplement 1). Their tumors were also more often smaller than 4 cm (32 of 55 [58.2%] vs 176 of 554 [31.8%]; *P* < .001) and located in the distal rectum (37 of 63 [58.7%] vs 131 of 581 [22.5%]; *P* < .001) and along the anterior rectal wall (20 of 57 [35.1%] vs 67 of 496 [13.6%]; *P* < .001).

More patients with PRC received any neoadjuvant therapy (NAT) (570 of 604 [94.4%]) compared with patients with SRC (33 of 64 [51.6%]) (Table 2). Contrary to treatment for patients with PRC, systemic chemotherapy only was the most common NAT among patients with SRC. Still, 13 of 64 patients with SRC (20.3%) received total NAT. A total of 11 patients with SRC treated with NAT until 2014 received chemotherapy only, whereas 14 of 22 patients treated after that year (63.6%) received re-irradiation treatment. Four of 33 patients (12.1%) did not complete planned NAT. One patient did not complete intended total NAT due to developing grade 3 scrotal and lower extremity swelling following RT and progression of disease during chemotherapy. One patient did not present to receive the last radiation fraction for unclear reasons. Two patients did not complete intended chemotherapy: 1 patient developed grade 3 diarrhea, and the other refused to receive further cycles. The toxic effect profile among patients with SRC who received NAT is summarized in eTable 1 in Supplement 1. Six of 33 patients (18.2%) developed grade 3 toxic effects, but there were no reported grade 4 or 5 toxic effects. The proportions of patients treated with NAT who were offered a watch-and-wait treatment plan were similar in both groups: 163 of 570 in the group with PRC (28.6%) compared with 7 of 33 in the group with SRC (21.2%). Among patients treated with surgery, more patients with SRC underwent abdominoperineal resection (6 of 12 [50.0%] vs 85 of 341 [24.9%]), pelvic exenteration (2 of 12 [16.7%] vs 5 of 341 [1.5%]), and local excision (1 of 12 [8.3%] vs 12 of 341 [3.5%]) (*P* < .001) compared with patients with PRC.

**Table 2.** Treatment Differences Between Patients With SRC and Those With PRC by Receipt of NAT

Treatment	Patients, No. (%)		*P* value
	PRC (n = 604)	SRC (n = 64)	
**Received NAT**			
Total	570 (94.4)	33 (51.6)	<.001
NAT type			
Chemotherapy only	59 (10.4)	19 (57.6)	<.001
Chemoradiation only	8 (1.4)	1 (3)	
Total NAT	503 (88.2)	13 (39.4)	
Primary treatment			
Nonoperative management	163 (28.6)	7 (21.2)	<.001
Other^a^	66 (11.6)	14 (42.4)	
Surgery			
Any	341 (59.8)	12 (36.4)	
Low anterior resection	239 (70.1)	3 (25.0)	<.001
Abdominoperineal resection	85 (24.9)	6 (50.0)	
Pelvic exenteration	5 (1.5)	2 (16.7)	
Local excision^b^	12 (3.5)	1 (8.3)	
**Did not receive NAT**			
Total	34 (5.6)	31 (48.4)	<.001
Primary treatment			
Other^a^	6 (17.6)	8 (25.8)	.55
Surgery			
Any	28 (82.4)	23 (74.2)	
Low anterior resection	16 (57.1)	6 (26.1)	.051
Abdominoperineal resection	2 (7.1)	7 (30.4)	
Pelvic exenteration	1 (3.6)	1 (4.4)	
Local excision^b^	9 (32.2)	9 (39.1)	

Abbreviations: NAT, neoadjuvant therapy; PRC, primary rectal cancer; SRC, secondary rectal cancer.

^a^ Patients who were not candidates for oncologic resection and were treated with either systemic therapy, palliative radiation, or bowel diversion or received no treatment.

^b^ Patients treated with local excision underwent transanal minimally invasive surgery, transanal excision, endoscopic submucosal dissection, or electrofulguration.

### Comparison of Oncologic Outcomes Between the Cohort With SRC and the Clinically Matched Cohort With PRC

**Figure 1.**
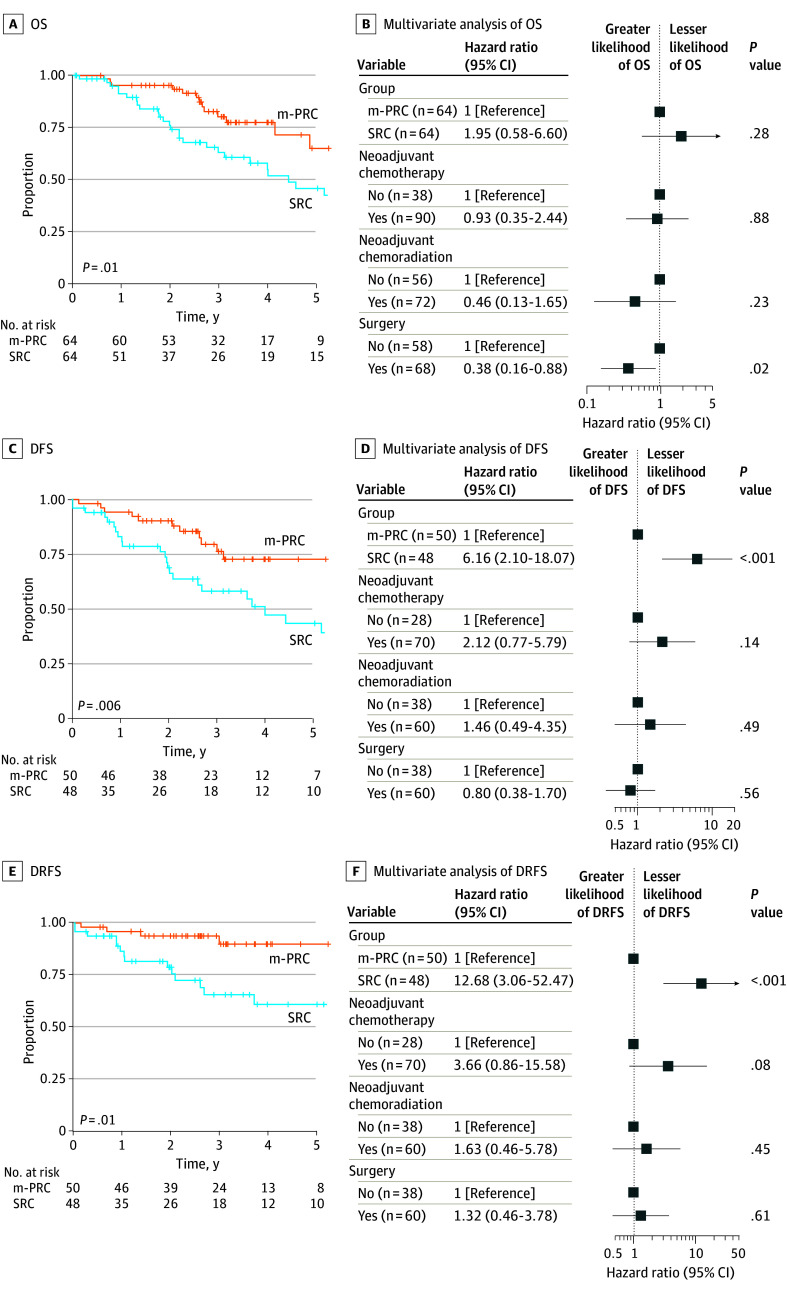
Comparison of Clinical Outcomes in Clinically Matched Patients With Primary Rectal Cancer (m-PRC) vs Those With Secondary Rectal Cancer (SRC) A, C, and E, The starting time point for all the curves is the time of rectal cancer diagnosis. *P* values were computed using a log-rank test. C and E, Patients with stage IV cancer were not included in the analyses. B, D, and F, Variables used in the analyses included treatment with neoadjuvant chemotherapy, neoadjuvant chemoradiotherapy, and surgical resection. DFS indicates disease-free survival; DRFS, distant recurrence–free survival; and OS, overall survival.

We verified that the clinical variables used for matching were not significantly different in the group with SRC (n = 64) and the clinically matched group of male patients with PRC (n = 64) (eFigure 2 and eTable 2 in Supplement 1). The group with SRC had shorter 5-year OS (45.7% vs 64.9%; *P* = .01) (Figure 1A), DFS (40.3% vs 71.2%; *P* = .006) (Figure 1C), and DRFS (60.8% vs 89.8%; *P* = .01) (Figure 1E) compared with clinically matched patients with PRC. No difference in local recurrence-free survival was observed between groups (92.6% vs 95.3%; *P* = .50) (eFigure 2C in Supplement 1). We performed multivariate survival analyses using a Cox proportional hazards regression model to investigate associations between groups and outcomes while accounting for differences in treatments (Figure 1B, D, and F). Secondary rectal cancer was associated with worse DFS (hazard ratio [HR], 6.16 [95% CI, 2.10-18.07]; *P* < .001) and DRFS (HR, 12.68 [95% CI, 3.06-52.47]; *P* < .001) but was not associated with OS (HR, 1.95 [95% CI, 0.58-6.60]; *P* = .28). Having surgery was the only factor associated with shorter OS (HR, 0.38 [95% CI, 0.16-0.88]; *P* = .02).

### Genomic Profile of Patients With SRC

We compared molecular features of 31 patients with SRC with available targeted DNA sequencing with both a background set of 541 patients with PRC and a clinically matched subset of 62 patients with PRC. We matched for the same clinical variables as in the aforementioned analysis; however, since previous genomic profiling of rectal cancer has not identified relevant molecular differences by sex,^22^ we did not match for sex in this propensity score matching model. This approach allowed us to use a 1:2 ratio for the matched cohort (62 clinically matched patients with PRC) (eFigure 1 and eTable 3 in Supplement 1 and eTable 11 in Supplement 2).

**Figure 2.**
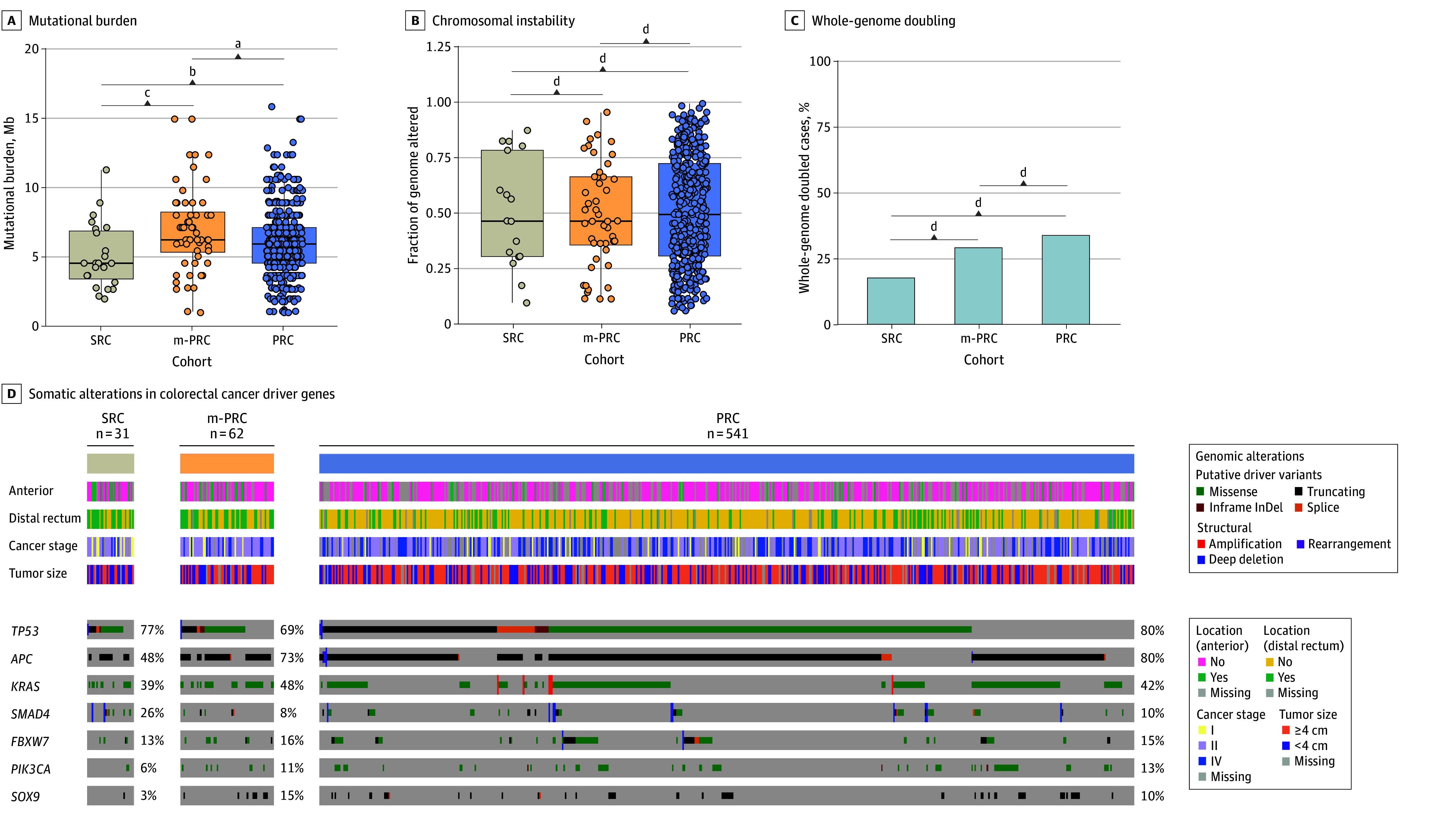
Comparison of Genomic Features Identified Through Targeted DNA Sequencing of Tumors Derived From Patients With Secondary Rectal Cancer (SRC), Patients With Primary Rectal Cancer (PRC), and Clinically Matched Patients With PRC (m-PRC) A and B, Horizontal bands in boxes represent medians; tops and bottoms of the boxes, IQRs; whiskers, ranges; and filled circles, samples. InDel indicates insertions and deletions. ^a^*P* = .01. ^b^*P* = .05. ^c^*P* = .006. ^d^No statistically significant difference.

None of the patients were microsatellite instable or had a polymerase epsilon hypermutation. TMB was lower in the SRC tumors (median, 4.4 [IQR, 3.2-6.7] per megabase [Mb]) compared with the PRC tumors (median, 5.8 [IQR, 4.4-7.0] per Mb) (*P* = .047) and clinically matched patients with PRC (median, 6.1 [IQR, 5.2-8.1] per Mb) (*P* = .006) (Figure 2A). By contrast, no significant differences were observed in terms of FGA (Figure 2B) or frequency of whole-genome duplication events (Figure 2C). We compared the frequency of alterations in previously identified rectal cancer driver genes^22^ that were altered in at least 10% of either cohort (Figure 2D and eTables 4 and 5 in Supplement 1). Inactivating somatic alterations in the adenomatous polyposis coli (*APC*; OMIM 611731) gene were significantly less frequent in patients with SRC (15 of 31 [48.4%]) compared with patients with PRC (432 of 541 [79.9%]) and clinically matched patients with PRC (45 of 62 [72.6%]) (*P* < .001 and *P* = .04, respectively). *SMAD4* (OMIM 600993) inactivation was more frequent in patients with SRC (8 of 31 [25.8%]) compared with patients with PRC (54 of 541 [10.0%]) (*P* = .01) and clinically matched patients with PRC (5 of 62 [8.1%]) (*P* = .03) (Figure 2D and eFigure 3 in Supplement 1). An analysis of alterations in oncogenic signaling pathways^30^ identified a higher rate of Wnt pathway alterations in the group with PRC compared with the group with SRC (450 of 541 [83.2%] vs 17 of 31 [54.8%]; *P* < .001), driven by the higher frequency of *APC* alterations (eFigure 3 and eTables 6 and 7 in Supplement 1). No major differences in genome-wide copy number patterns and arm-level changes were observed across cohorts (eFigure 3 and eTable 8 in Supplement 1).

### Mutational Analyses of SRC Tumors Using WES

**Figure 3.**
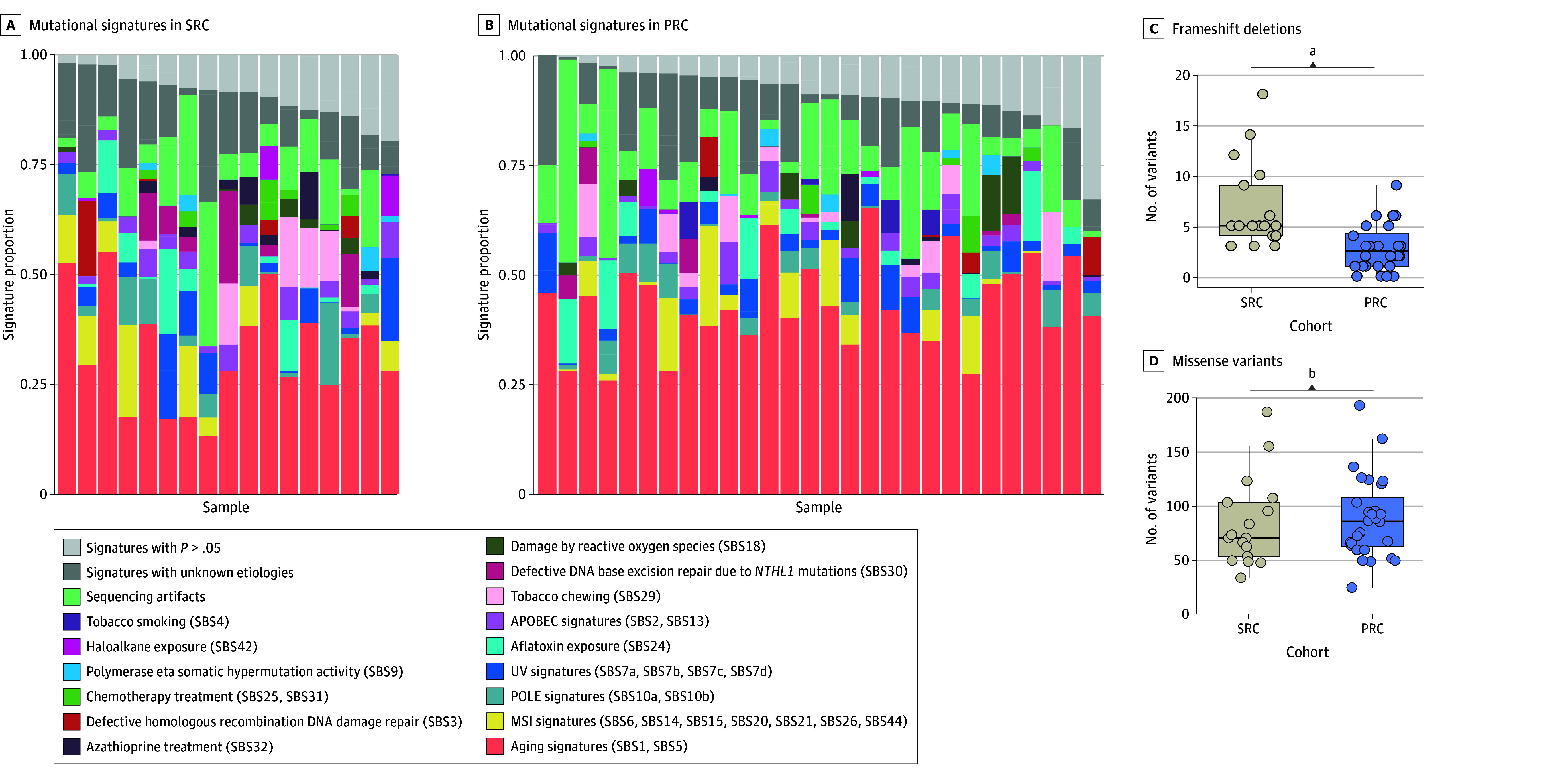
Analysis of Mutational Patterns Using Whole-Exome Sequencing (WES) From Patients With Primary Rectal Cancer (PRC) or Secondary Rectal Cancer (SRC) A and B, Mutational signatures from the COSMIC catalog for patients in the cohorts with SRC and PRC with available WES data. Panels show signature decomposition per sample. C and D, Number of variants, by type, identified using WES of specimens from the cohorts with SRC and PRC. APOBEC indicates apolipoprotein B mRNA editing catalytic polypeptide-like; MSI, microsatellite instability; POLE, DNA polymerase epsilon, catalytic subunit. ^a^*P* ≤ .001. ^b^No statistically significant difference.

Mutational signatures in patients with SRC with available WES data (n = 17) were compared with those observed in patients with PRC (n = 28) (Figure 3A and B). The most common signature observed across samples was SBS1, a clocklike signature that has been associated with aging. No significant differences were observed in terms of any of the signatures from the COSMIC catalog between the groups with SRC and PRC, including the SBS6 and SBS15 signatures associated with defective mismatch repair (eTable 9 in Supplement 1).

Tumors from patients with SRC had more frameshift deletions than tumors from patients with PRC (median, 5.0 [IQR, 4.0-9.0] vs 2.5 [IQR, 1.0-4.2] variants; *P* < .001) (Figure 3C). Tumors from patients with SRC also had more inframe deletions (median, 1.0 [IQR, 1.0-2.0] vs 1.0 [IQR, 0.0-1.0]; *P* = .03) and splice site variants (median, 2.0 [IQR, 1.0-3.0] vs 1.0 [IQR, 0.0-2.0]); *P* = .02) (eFigure 4 in Supplement 1). By contrast, the total number of variants was similar between the 2 groups (median, 122.0 [IQR, 105.0-176.0] vs 138.0 [IQR, 107.5-179.0]; *P* = .72), and no significant differences were observed in the number of missense variants (median, 70.0 [IQR, 53.0-103.0] vs 85.5 [IQR, 62.0-107.2]; *P* = .54) (Figure 3D and eFigure 4 in Supplement 1).

## Discussion

Data from this case-control study showed that patients developing SRC after RT for PC were older and had smaller, earlier-stage tumors located more often in the anterior rectal wall and closer to the anal verge compared with patients with PRC. Patients with SRC were less likely to receive neoadjuvant chemoradiation, systemic chemotherapy, or surgery with curative intent. They were also at a greater risk of distant metastasis and had shorter DFS and OS. While the differences in outcomes could be attributed to differences in treatment, SRC status was an independent factor associated with distant metastasis and lower DFS but not OS. Having surgery was the only independent factor associated with OS in multivariable analysis. Our data suggest an increased comfort in re-irradiation treatment of patients with SRC over time, in which patients started receiving re-irradiation treatment only after 2014. None of the 11 patients treated with NAT for rectal cancer until 2014 received RT, whereas 14 of 22 patients with SRC treated after that year (63.6%) received re-irradiation treatment.

Previous studies using institutional case series have reported higher rates of recurrence and/or decreased survival among patients with SRC after the diagnosis of PC.^31,32,33,34^ Yang et al^5^ found that patients with primary pelvic tumors treated with RT were at increased risk of developing SRC, particularly when they received both EBRT and BRT. They also found shorter OS for patients with SRC compared with clinically matched patients with PRC. Rombouts et al^9^ also found a higher incidence of SRC among patients with pelvic cancer treated with RT in the Netherlands Cancer Registry. Patients with pelvic cancer treated without RT had similar risk of SRC compared with the general population, and OS after treatment for SRC was significantly shorter than OS after treatment for PRC.^9^

Prior to our study, the molecular profile of SRC had remained unexplored with the exception of a case series of 5 patients with SRC after RT for cervical cancer.^35^ In our study, we found that SRC tumors had lower TMB than PRC tumors in clinically matched patients, without a significant difference in FGA. Somatic *APC* alterations and Wnt pathway alterations were less frequent in the group with SRC, while *SMAD4* alterations were more common. Since *APC* wild-type status and *SMAD4* alterations have been linked to more aggressive tumors,^36,37^ these data could contribute to explaining the differences in outcomes between the group with SRC and the group with PRC.

Previous studies have reported an abundance of deletions and balanced inversions in radiation-associated tumors compared with radiation-naive tumors^17,38^ as well as in paired primary and recurrent tumors treated with RT.^39^ A possible mechanism to explain those differences is that RT induces a rise in double-stranded breaks within the DNA and that faulty repair of these breaks results in a higher incidence of those types of variants. We observed a higher frequency of frameshift deletions, inframe deletions, and splice site variants in SRC tumors than in PRC tumors despite lower TMB. Compared with single-nucleotide variants, frameshift insertions and deletions cause greater changes in downstream reading frames that can result in more potent neoantigens, enhanced immunogenicity, and ultimately improved responses to immune checkpoint inhibition.^40,41^ Based on these findings, it is tempting to speculate that patients with SRC might be good candidates for immunotherapy, which could provide an additional treatment option for this patient population.

### Limitations

This study has limitations. It was a retrospective study of patients accrued over almost 3 decades. This design entails inevitable heterogeneity in terms of diagnosis, treatment, and surveillance of both prostate cancer and rectal adenocarcinoma. Similarly, changes in the dose, delivery, and available radiation modalities used to treat both prostate and rectal cancers may have led to cross-patient variability. Furthermore, cases of SRC in the present study could have included instances of sporadic rectal cancer not directly caused by previous radiation; however, we expect these to be few, and the observed significant differences in clinical and genomic variables support the group with SRC being a distinct population. In addition, our genomic analyses were limited by small sample sizes, which resulted in restricted statistical power. Future studies using larger, independent cohorts of patients with SRC will be needed to validate our findings.

## Conclusions

In this case-control study, patients with SRC treated with RT for PC had worse survival outcomes than clinically matched patients with PRC. Secondary rectal cancer tumors had distinct molecular features, including lower mutational burden, lower frequency of *APC* alterations, higher rates of *SMAD4* inactivation, and increased rates of frameshift and inframe deletions. These insights can be used to improve the clinical management of patients with SRC.

## Notes

Supplement 1. **eFigure 1.** Sample selection and overview of cohorts used for analysis **eFigure 2.** Comparison of clinical features and outcomes between SRC and PRC patients **eTable 1.** Tolerance and toxicity profile of SRC patients treated with neoadjuvant treatment **eTable 2.** PSM (1:1 ratio) of m-PRC and SRC groups used for comparison of oncologic outcomes **eTable 3.** PSM (2:1 ratio) of m-PRC and SRC groups used for comparison of sample level features and frequencies of driver alterations **eTable 4.** Gene level comparison between SRC and PRC cohort **eTable 5.** Gene level comparison between SRC and m-PRC cohort **eFigure 3.** Extended results from genomic and clinical comparisons **eTable 6.** Pathway level comparison between SRC and PRC cohort **eTable 7.** Pathway level comparison between SRC and m-PRC cohort **eTable 8.** A-D, Arm level comparison between SRC and m-PRC cohort **eTable 9.** Mutational signature comparison from WES data (SRC vs PRC) **eFigure 4.** Extended results of mutational patterns using WES from SRC and PRC patients

Supplement 2. **eTable 10.** Clinical and histopathological data per patient **eTable 11.** Additional information for patients sequenced with MSK-IMPACT **eTable 12.** Additional information for patients sequenced with WES

Supplement 3.
Data Sharing Statement
