# Supplementary material for: Clinical and Genomic Characterization of Secondary Rectal Cancer After Radiotherapy for Prostate Cancer
Source: JAMA Netw Open. 2025 Mar 18;8(3):e251039. doi: 10.1001/jamanetworkopen.2025.1039 (PMC11920846; doi:10.1001/jamanetworkopen.2025.1039)
Supplement: Supplement 1. — eFigure 1. Sample selection and overview of cohorts used for analysis eFigure 2. Comparison of clinical features and outcomes between SRC and PRC patients eTable 1. Tolerance and toxicity profile of SRC patients treated with neoadjuvant treatment eTable 2. PSM (1:1 ratio) of m-PRC and SRC groups used for comparison of oncologic outcomes eTable 3. PSM (2:1 ratio) of m-PRC and SRC groups used for comparison of sample level features and frequencies of driver alterations eTable 4. Gene level comparison between SRC and PRC cohort eTable 5. Gene level comparison between SRC and m-PRC cohort eFigure 3. Extended results from genomic and clinical comparisons eTable 6. Pathway level comparison between SRC and PRC cohort eTable 7. Pathway level comparison between SRC and m-PRC cohort eTable 8. A-D, Arm level comparison between SRC and m-PRC cohort eTable 9. Mutational signature comparison from WES data (SRC vs PRC) eFigure 4. Extended results of mutational patterns using WES from SRC and PRC patients [file jamanetwopen-e251039-s001.pdf]

Supplementary Online Content

Omer DM, Shah F, Luthra A, et al. Clinical and genomic characterization of secondary rectal cancer after radiotherapy for prostate cancer. *JAMA Netw Open*. 2025;8(3):e251039. doi:10.1001/jamanetworkopen.2025.1039

eFigure 1. Sample selection and overview of cohorts used for analysis
eFigure 2. Comparison of clinical features and outcomes between SRC and PRC patients
eTable 1. Tolerance and toxicity profile of SRC patients treated with neoadjuvant treatment
eTable 2. PSM (1:1 ratio) of m-PRC and SRC groups used for comparison of oncologic outcomes
eTable 3. PSM (2:1 ratio) of m-PRC and SRC groups used for comparison of sample level features and frequencies of driver alterations
eTable 4. Gene level comparison between SRC and PRC cohort
eTable 5. Gene level comparison between SRC and m-PRC cohort
eFigure 3. Extended results from genomic and clinical comparisons
eTable 6. Pathway level comparison between SRC and PRC cohort
eTable 7. Pathway level comparison between SRC and m-PRC cohort
eTable 8. A-D, Arm level comparison between SRC and m-PRC cohort
eTable 9. Mutational signature comparison from WES data (SRC vs. PRC)
eFigure 4. Extended results of mutational patterns using WES from SRC and PRC patients

This supplementary material has been provided by the authors to give readers additional information about their work.

© 2025 Omer DM. *JAMA Network Open*.

eFigure 1. Sample selection and overview of cohorts used for analysis

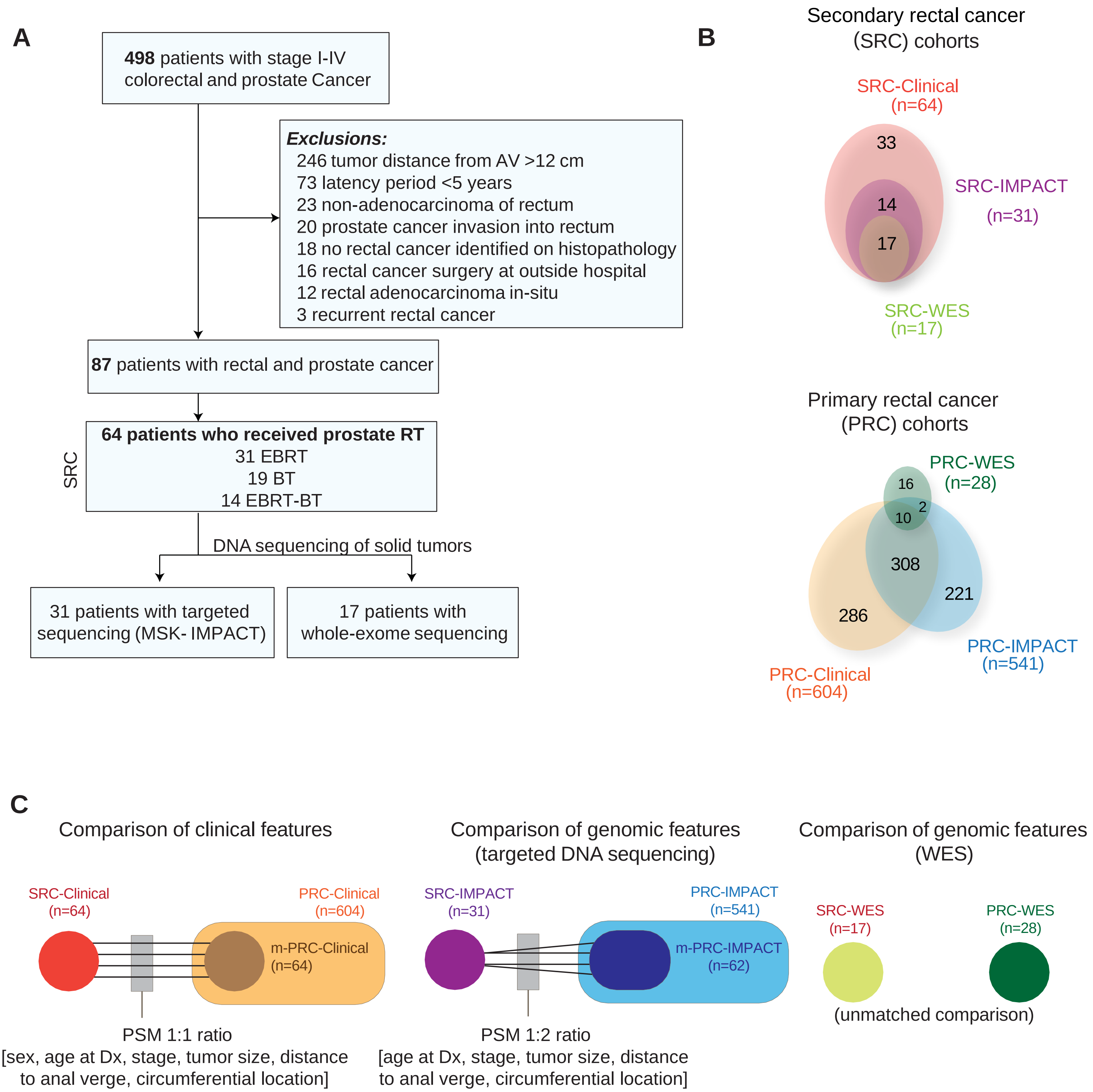

A. CONSORT-like diagram summarizing patient inclusion and exclusion criteria. B. Venn diagrams showing overlaps between the different SRC and PRC cohorts. C. Schematic diagrams summarizing key features of the cohorts used for comparison of clinical features (left), comparison of genomic features from targeted sequencing (middle) and comparison of genomic features from WES (right).

© 2025 Omer DM. *JAMA Network Open*.

eFigure 2. Comparison of clinical features and outcomes between SRC and PRC patients

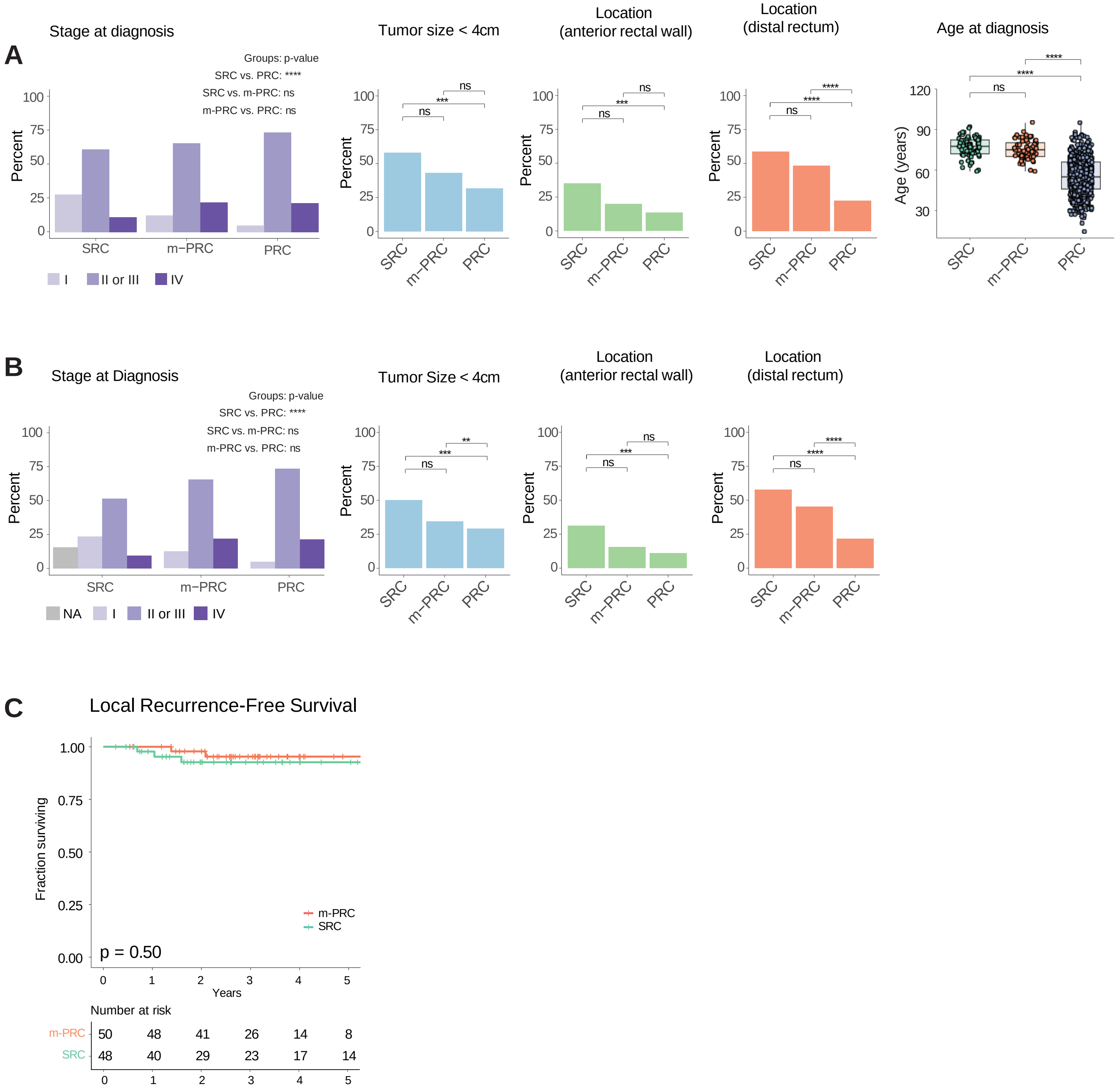

A. Stage at diagnosis, tumor size, circumferential tumor location, distal rectum location and age at diagnosis for patients in the SRC, m-PRC and PRC cohorts. B. Stage at diagnosis, tumor size, circumferential tumor location and distal rectum location for patients in the SRC, m-PRC and PRC cohorts, including unknown cases. p-values were calculated using two-sided Fisher's exact test.
C. Comparison of local recurrence-free survival between the SRC group and m-PRC groups. The starting time point for all the curves is time of rectal cancer diagnosis. p-values were computed using a log-rank test. Stage IV patients are not included.
Abbreviations- n.s: not significant, p>0.05, * p≤0.05, ** p≤0.01, *** p≤0.001, **** p≤0.0001.

© 2025 Omer DM. *JAMA Network Open*.

**eTable 1. Tolerance and toxicity profile of SRC patients treated with neoadjuvant treatment**			
	**Total neoadjuvant therapy, (n=13)**	**Chemotherapy only, (n=19)**	**Chemoradiation only, (n=1)**
Completed intended treatment, n (%)			
Chemotherapy	13 (100)	17 (89.5)	-
CRT	11 (84.6)	-	1 (100)
Grade 1 toxicity	6 (46.2)	7 (36.8)	1 (100)
Grade 2 toxicity	10 (76.9)	7 (36.8)	0
Grade 3 toxicity	3 (23.1)	3 (15.8)	0
Grade 4 and 5 toxicity	0	0	0
CRT related grade 3 toxicity			
Lower extremity swelling	1	-	0
Scrotal swelling	1	-	0
Diarrhea	1	-	0
Chemotherapy related grade 3 toxicity			
Neutropenia	1	0	-
Fatigue	0	1	-
Diarrhea	0	2	-

Summary of completion of intended neoadjuvant treatment and toxicity profile of SRC patients treated with neoadjuvant treatment for rectal cancer.

© 2025 Omer DM. *JAMA Network Open*.

**eTable 2. PSM (1:1 ratio) of m-PRC and SRC groups used for comparison of oncologic outcomes**					
**Clinical Characteristics**	**Patients No. (%)**			**p-value (SRC vs PRC)**	**p-value (SRC vs m-PRC)**
	**PRC (n = 604)**	**SRC (n = 64)**	**m-PRC (n = 64)**		
**Median age at rectal cancer diagnosis, years (IQR)**	55 (46 - 66)	78 (72 - 82)	75 (70 - 80)	<0.001	0.1
**cTNM stage**	604	54	64	<0.001	0.067
Local	30 (5.0)	15 (27.8)	8 (12.5)		
Locally advanced	445 (73.7)	33 (61.1)	42 (65.6)		
Metastatic	129 (21.3)	6 (11.1)	14 (21.9)		
Unknown	0	10	0	NA	NA
**Tumor size < 4cm**	554	55	51	<0.001	0.2
No	378 (68.2)	23 (41.8)	29 (56.9)		
Yes	176 (31.8)	32 (58.2)	22 (43.1)		
Unknown	50	9	13	NA	NA
**Anterior Tumor Location**	496	57	50	<0.001	0.09
No	429 (86.5)	37 (64.9)	40 (80.0)		
Yes	67 (13.5)	20 (35.1)	10 (20.0)		
Unknown	108	7	14	NA	NA
**Distal Tumor Location**	581	63	60	<0.001	0.3
No	450 (77.5)	26 (41.3)	31 (51.7)		
Yes	131 (22.5)	37 (58.7)	29 (48.3)		
Unknown	23	1	4	NA	NA

p-values were calculated using two- sided exact Fisher’s test.
Abbreviations- PSM, Propensity Score Matching, IQR, Interquartile range, No. Number, NA, Not Applicable.

© 2025 Omer DM. *JAMA Network Open*.

eTable 3. PSM (2:1 ratio) of m-PRC and SRC groups used for comparison of sample level features and frequencies of driver alterations

**Clinical Characteristics**	**Patients No. (%)**		**p-value**
	**m-PRC (n = 62)**	**SRC (n = 31)**	
**Median age at rectal cancer diagnosis, years (IQR)**	75 (69 - 80)	77 (72 - 83)	0.3
**cTNM stage**	61	29	0.2
Local	7 (11.5)	8 (27.6)	
Locally advanced	41 (67.2)	17 (58.6)	
Metastatic	13 (21.3)	4 (13.8)	
Unknown	1	2	NA
**Tumor size < 4cm**	57	29	0.2
No	33 (57.9)	12 (41.4)	
Yes	24 (42.1)	17 (58.6)	
Unknown	5	2	NA
**Anterior Tumor Location**	51	29	0.5
No	37 (72.5)	18 (62.0)	
Yes	14 (27.5)	11 (38.0)	
Unknown	11	2	NA
**Distal Tumor Location**	60	31	0.5
No	28 (46.7)	12 (38.7)	
Yes	32 (53.3)	19 (61.3)	
Unknown	2	0	NA

p-values were calculated using two-sided exact Fisher's test.
Abbreviations- PSM, Propensity Score Matching, IQR, Interquartile range, No. Number, NA, Not Applicable.

© 2025 Omer DM. *JAMA Network Open*.

eTable 4. Gene level comparison between SRC and PRC cohort

Gene	SRC Altered (n)	SRC WT (n)	SRC Altered Freq (%)	PRC Altered (n)	PRC WT (n)	PRC Altered Freq (%)	p-value	p-value corrected
APC	15	16	48.4	432	109	79.9	0.00	0.001
SMAD4	8	23	25.8	54	487	10.0	0.01	0.04
PIK3CA	2	29	6.5	71	470	13.1	0.41	0.71
SOX9	1	30	3.2	56	485	10.4	0.35	0.71
TP53	24	7	77.4	433	108	80.0	0.65	0.91
KRAS	12	19	38.7	225	316	41.6	0.85	0.99
FBXW7	4	27	12.9	82	459	15.2	1.00	1.00

Gene level comparison between SRC and PRC cohort. p-values were calculated using two-sided Fisher's exact test. Multiple hypothesis was performed using Benjamini-Hochberg Procedure on previously identified rectal cancer driver genes with 10% alteration frequency in any group.
Abbreviations- WT, Wild Type

© 2025 Omer DM. *JAMA Network Open*.

eTable 5. Gene level comparison between SRC and m-PRC cohort							
**Gene**	**SRC Altered (n)**	**SRC WT (n)**	**SRC Altered Freq (%)**	**m-PRC Altered (n)**	**m-PRC WT (n)**	**m-PRC Altered Freq (%)**	**p-value**
SMAD4	8	23	25.8	5	57	8.1	0.03
APC	15	16	48.4	45	17	72.6	0.04
ATM	2	29	6.5	0	62	0.0	0.11
SOX9	1	30	3.2	9	53	14.5	0.16
CDK8	2	29	6.5	1	61	1.6	0.26
MYC	2	29	6.5	1	61	1.6	0.26
ERBB2	0	31	0.0	4	58	6.5	0.30
TP53	24	7	77.4	43	19	69.4	0.47
ARID1A	2	29	6.5	8	54	12.9	0.49
KRAS	12	19	38.7	30	32	48.4	0.51
ERBB3	0	31	0.0	2	60	3.2	0.55
PTEN	3	28	9.7	4	58	6.5	0.68
PIK3CA	2	29	6.5	7	55	11.3	0.71
FBXW7	4	27	12.9	10	52	16.1	0.77
ARID1B	0	31	0.0	0	62	0.0	1.00
BRAF	1	30	3.2	3	59	4.8	1.00
AMER1	1	30	3.2	2	60	3.2	1.00
NRAS	1	30	3.2	2	60	3.2	1.00
TCF7L2	2	28	6.7	4	55	6.8	1.00

Gene level comparison between SRC and m-PRC cohort. p-values were calculated using two-sided Fisher’s exact test. Abbreviations- WT, Wild Type

© 2025 Omer DM. *JAMA Network Open*.

eFigure 3. Extended results from genomic and clinical comparisons

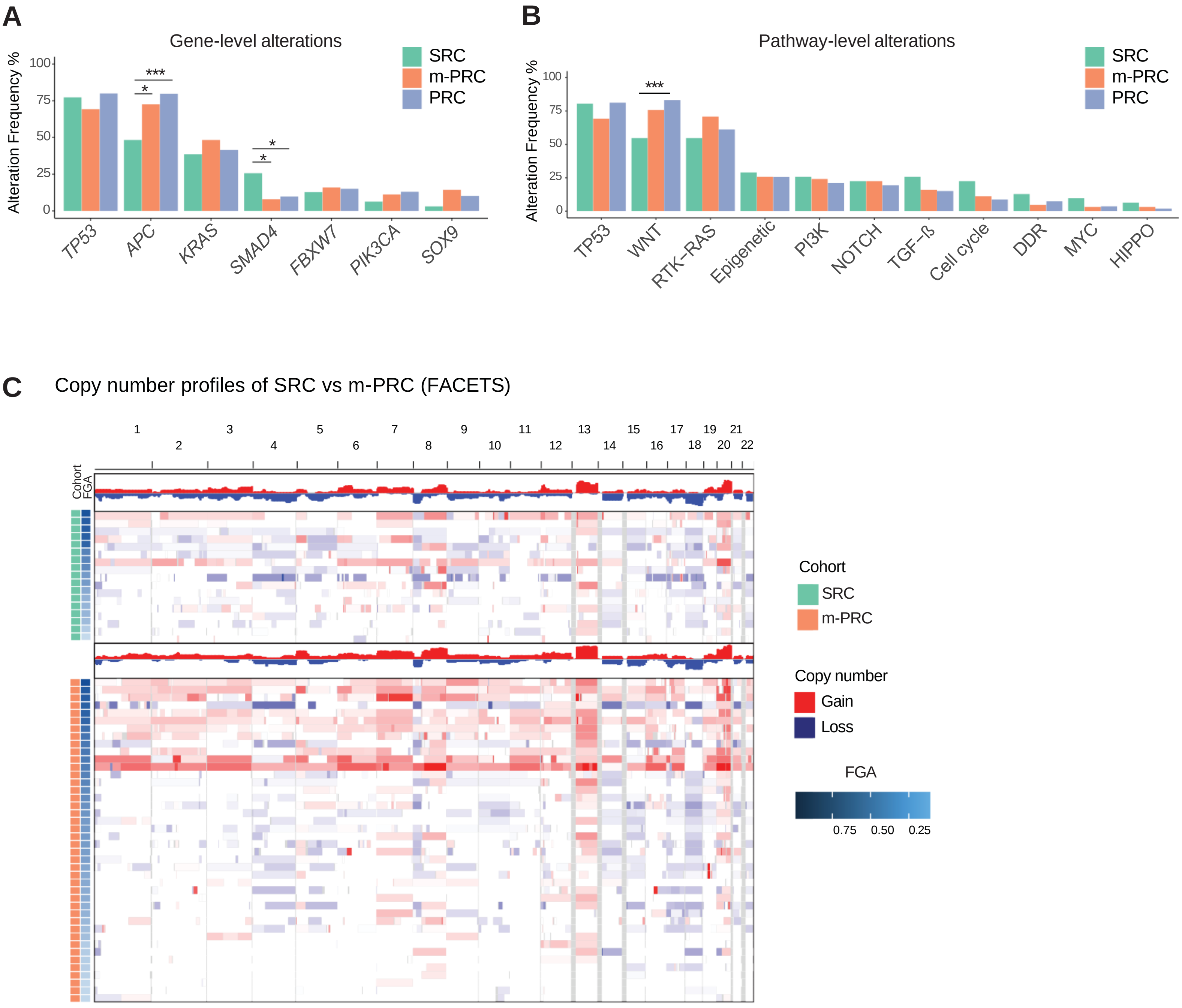

A. Comparison of gene alteration frequencies. B. Comparison of pathway alteration frequencies. C. Genome-wide view of copy number profiles for specimens in the SRC and the PRC groups. p-values were calculated using two-sided exact Fisher's test
Abbreviations- FGA: Fraction Genome Altered, n.s: not significant, p>0.05, * p≤0.05, ** p≤0.01, *** p≤0.001, **** p≤0.0001.

© 2025 Omer DM. *JAMA Network Open*.

eTable 6. Pathway level comparison between SRC and PRC cohort								
**Gene**	**SRC Altered (n)**	**SRC WT (n)**	**SRC Altered Freq (%)**	**PRC Altered (n)**	**PRC WT (n)**	**PRC Altered Freq (%)**	**p-value**	**p-value corrected**
WNT	17	14	54.8	450	91	83.2	0.00	0.00
Cell cycle	7	24	22.6	48	493	8.9	0.02	0.12
HIPPO	2	29	6.5	11	530	2.0	0.15	0.34
MYC	3	28	9.7	20	521	3.7	0.12	0.34
TGF-Beta	8	23	25.8	82	459	15.2	0.13	0.34
DDR	4	27	12.9	40	501	7.4	0.29	0.53
PI3K	8	23	25.8	114	427	21.1	0.50	0.74
Epigenetic	9	22	29.0	139	402	25.7	0.68	0.74
RTK-RAS	17	14	54.8	331	210	61.2	0.57	0.74
NOTCH	7	24	22.6	105	436	19.4	0.64	0.74
TP53	25	6	80.6	440	101	81.3	1.00	1.00

p-values were calculated using two-sided Fisher's exact test. Multiple hypothesis testing was performed using Benjamini-Hochberg Procedure.
Abbreviations- WT, Wild Type

© 2025 Omer DM. *JAMA Network Open*.

eTable 7. Pathway level comparison between SRC and m-PRC cohort							
**Gene**	**SRC Altered (n)**	**SRC WT (n)**	**SRC Altered Freq (%)**	**m-PRC Altered (n)**	**m-PRC WT (n)**	**m-PRC Altered Freq (%)**	**p-value**
WNT	17	14	54.8	47	15	75.8	0.06
RTK-RAS	17	14	54.8	44	18	71.0	0.17
DDR	4	27	12.9	3	59	4.8	0.22
Cell cycle	7	24	22.6	7	55	11.3	0.22
TGF-Beta	8	23	25.8	10	52	16.1	0.28
TP53	25	6	80.6	43	19	69.4	0.32
MYC	3	28	9.7	2	60	3.2	0.33
HIPPO	2	29	6.5	2	60	3.2	0.60
Epigenetic	9	22	29.0	16	46	25.8	0.81
PI3K	8	23	25.8	15	47	24.2	1.00
NOTCH	7	24	22.6	14	48	22.6	1.00
NRF2	0	31	0.0	0	62	0.0	1.00

p-values were calculated using two-sided Fisher's exact test.
Abbreviations- WT, Wild Type

© 2025 Omer DM. *JAMA Network Open*.

eTable 8. Arm level comparison between SRC and m-PRC cohort<br>A. Amplifications								
**Arm level Amplifications**	**m-PRC amp (n)**	**m-PRC No-amp (n)**	**SRC amp (n)**	**SRC No-amp (n)**	**m-PRC amp Freq (%)**	**SRC amp Freq (%)**	**p-value**	**Corrected p-value**
6p	0	47	1	16	0.0	5.9	0.27	1
13q	6	41	0	15	12.8	0.0	0.32	1
1q	1	41	0	15	2.4	0.0	1.00	1
1p	0	35	0	12	0.0	0.0	1.00	1
2p	0	45	0	16	0.0	0.0	1.00	1
2q	0	42	0	16	0.0	0.0	1.00	1
3p	0	47	0	15	0.0	0.0	1.00	1
3q	0	43	0	13	0.0	0.0	1.00	1
4p	0	49	0	16	0.0	0.0	1.00	1
4q	0	39	0	13	0.0	0.0	1.00	1
5p	1	44	0	16	2.2	0.0	1.00	1
5q	0	36	0	10	0.0	0.0	1.00	1
6q	0	43	0	15	0.0	0.0	1.00	1
7p	2	45	0	15	4.3	0.0	1.00	1
7q	1	43	0	16	2.3	0.0	1.00	1
8p	0	47	0	15	0.0	0.0	1.00	1
8q	3	43	0	14	6.5	0.0	1.00	1
9p	0	41	0	15	0.0	0.0	1.00	1
9q	0	46	0	15	0.0	0.0	1.00	1
10p	0	49	0	15	0.0	0.0	1.00	1
10q	0	45	0	12	0.0	0.0	1.00	1
11p	0	47	0	17	0.0	0.0	1.00	1
11q	0	46	0	15	0.0	0.0	1.00	1
12p	0	37	0	16	0.0	0.0	1.00	1
12q	0	43	0	13	0.0	0.0	1.00	1
14q	0	47	0	18	0.0	0.0	1.00	1
15q	0	45	0	16	0.0	0.0	1.00	1
16p	0	48	0	16	0.0	0.0	1.00	1
16q	0	44	0	16	0.0	0.0	1.00	1
17p	0	47	0	15	0.0	0.0	1.00	1
17q	0	43	0	14	0.0	0.0	1.00	1
18p	0	48	0	15	0.0	0.0	1.00	1
18q	0	48	0	16	0.0	0.0	1.00	1
19p	1	41	0	12	2.4	0.0	1.00	1
19q	1	45	0	15	2.2	0.0	1.00	1
20p	6	44	2	15	12.0	11.8	1.00	1
20q	7	40	2	16	14.9	11.1	1.00	1
21q	0	47	0	17	0.0	0.0	1.00	1
22q	0	48	0	16	0.0	0.0	1.00	1

p-values were calculated using two-sided Fisher's exact test. Multiple hypothesis was performed using Benjamini-Hochberg Procedure.
Abbreviations- Amp, Amplifications, No-amp, No Amplifications, Freq, Frequency

© 2025 Omer DM. *JAMA Network Open*.

eTable 8. Arm level comparison between SRC and m-PRC cohort
B. Amplifications & Gains

Arm level Amp & Gain	m-PRC amp & gain (n)	m-PRC No-amp or gain (n)	SRC amp & gain (n)	SRC No-amp or gain (n)	m-PRC amp Freq (%)	SRC amp Freq (%)	p-value	Corrected p-value
12q	8	35	0	13	18.6	0.0	0.18	1
7p	19	28	3	12	40.4	20.0	0.22	1
20p	27	23	6	11	54.0	35.3	0.26	1
5p	13	32	2	14	28.9	12.5	0.31	1
11p	5	42	0	17	10.6	0.0	0.31	1
16p	5	43	0	16	10.4	0.0	0.32	1
11q	6	40	0	15	13.0	0.0	0.32	1
1q	6	36	0	15	14.3	0.0	0.32	1
20q	32	15	10	8	68.1	55.6	0.39	1
4q	1	38	1	12	2.6	7.7	0.44	1
1p	1	34	1	11	2.9	8.3	0.45	1
7q	16	28	4	12	36.4	25.0	0.54	1
13q	28	19	7	8	59.6	46.7	0.55	1
17q	4	39	0	14	9.3	0.0	0.56	1
8p	4	43	0	15	8.5	0.0	0.56	1
19p	4	38	0	12	9.5	0.0	0.56	1
6q	4	39	2	13	9.3	13.3	0.64	1
3q	7	36	1	12	16.3	7.7	0.67	1
3p	7	40	1	14	14.9	6.7	0.67	1
17p	1	46	0	15	2.1	0.0	1.00	1
18q	1	47	0	16	2.1	0.0	1.00	1
21q	2	45	1	16	4.3	5.9	1.00	1
22q	1	47	0	16	2.1	0.0	1.00	1
2p	2	43	1	15	4.4	6.3	1.00	1
2q	2	40	1	15	4.8	6.3	1.00	1
4p	2	47	1	15	4.1	6.3	1.00	1
5q	3	33	0	10	8.3	0.0	1.00	1
6p	8	39	3	14	17.0	17.6	1.00	1
8q	14	32	4	10	30.4	28.6	1.00	1
9p	4	37	1	14	9.8	6.7	1.00	1
9q	4	42	1	14	8.7	6.7	1.00	1
10p	2	47	0	15	4.1	0.0	1.00	1
10q	0	45	0	12	0.0	0.0	1.00	1
12p	7	30	3	13	18.9	18.8	1.00	1
14q	2	45	1	17	4.3	5.6	1.00	1
15q	2	43	1	15	4.4	6.3	1.00	1
16q	3	41	1	15	6.8	6.3	1.00	1
18p	2	46	0	15	4.2	0.0	1.00	1
19q	4	42	1	14	8.7	6.7	1.00	1

p-values were calculated using two-sided Fisher's exact test. Multiple hypothesis was performed using Benjamini-Hochberg Procedure.
Abbreviations- amp & gain, Amplifications and Gains, No-amp or gain, No Amplifications or Gains, Freq, Frequency

© 2025 Omer DM. *JAMA Network Open*.

eTable 8. Arm level comparison between SRC and m-PRC cohort<br>C. Deletions								
**Arm level Deletions**	**m-PRC del (n)**	**m-PRC No-del (n)**	**SRC del (n)**	**SRC No-del (n)**	**m-PRC del Freq (%)**	**SRC del Freq (%)**	**p-value**	**Corrected p-value**
22q	0	18	1	8	0.0	11.1	0.33	1
18p	0	9	1	4	0.0	20.0	0.36	1
18q	0	9	1	4	0.0	20.0	0.36	1
1p	0	21	0	6	0.0	0.0	1	1
1q	0	33	0	9	0.0	0.0	1	1
2p	1	31	0	13	3.1	0.0	1	1
2q	0	31	0	10	0.0	0.0	1	1
3p	0	34	0	7	0.0	0.0	1	1
3q	0	32	0	7	0.0	0.0	1	1
4p	0	21	0	8	0.0	0.0	1	1
4q	0	15	0	5	0.0	0.0	1	1
5p	0	32	0	12	0.0	0.0	1	1
5q	0	16	0	5	0.0	0.0	1	1
6p	0	36	0	11	0.0	0.0	1	1
6q	0	32	0	10	0.0	0.0	1	1
7p	0	42	0	13	0.0	0.0	1	1
7q	0	39	0	14	0.0	0.0	1	1
8p	1	19	0	7	5.0	0.0	1	1
8q	0	31	0	10	0.0	0.0	1	1
9p	0	24	0	9	0.0	0.0	1	1
9q	0	27	0	10	0.0	0.0	1	1
10p	0	23	0	9	0.0	0.0	1	1
10q	0	21	0	8	0.0	0.0	1	1
11p	0	33	0	11	0.0	0.0	1	1
11q	0	33	0	9	0.0	0.0	1	1
12p	0	27	0	11	0.0	0.0	1	1
12q	0	31	0	9	0.0	0.0	1	1
13q	0	39	0	14	0.0	0.0	1	1
14q	0	15	0	6	0.0	0.0	1	1
15q	0	12	0	8	0.0	0.0	1	1
16p	0	31	0	10	0.0	0.0	1	1
16q	0	26	0	10	0.0	0.0	1	1
17p	0	13	0	4	0.0	0.0	1	1
17q	0	19	0	8	0.0	0.0	1	1
19p	0	27	0	6	0.0	0.0	1	1
19q	0	27	0	10	0.0	0.0	1	1
20p	1	41	0	10	2.4	0.0	1	1
20q	0	44	0	14	0.0	0.0	1	1
21q	1	24	0	7	4.0	0.0	1	1

p-values were calculated using two-sided Fisher's exact test. Multiple hypothesis was performed using Benjamini-Hochberg Procedure.
Abbreviations- del, Deletions, No-del, No Deletions, Freq, Frequency

© 2025 Omer DM. *JAMA Network Open*.

eTable 8. Arm level comparison between SRC and m-PRC cohort
D. Deletions and Loss

Arm level Del and Loss	m-PRC del & loss (n)	m-PRC No-del or loss (n)	SRC del & loss (n)	SRC No-del or loss (n)	m-PRC del & loss Freq (%)	SRC del & loss Freq (%)	p-value	Corrected p-value
20q	3	44	4	14	6.4	22.2	0.09	1
20p	10	40	7	10	20.0	41.2	0.11	1
3p	13	34	8	7	27.7	53.3	0.12	1
15q	33	12	8	8	73.3	50.0	0.12	1
3q	11	32	6	7	25.6	46.2	0.18	1
1q	9	33	6	9	21.4	40.0	0.19	1
10q	24	21	4	8	53.3	33.3	0.33	1
2p	15	30	3	13	33.3	18.8	0.35	1
6p	11	36	6	11	23.4	35.3	0.35	1
13q	8	39	1	14	17.0	6.7	0.43	1
19p	15	27	6	6	35.7	50.0	0.50	1
2q	11	31	6	10	26.2	37.5	0.52	1
11q	13	33	6	9	28.3	40.0	0.52	1
17q	24	19	6	8	55.8	42.9	0.54	1
10p	26	23	6	9	53.1	40.0	0.56	1
1p	14	21	6	6	40.0	50.0	0.74	1
6q	11	32	5	10	25.6	33.3	0.74	1
12p	10	27	5	11	27.0	31.3	0.75	1
9q	19	27	5	10	41.3	33.3	0.76	1
19q	19	27	5	10	41.3	33.3	0.76	1
11p	14	33	6	11	29.8	35.3	0.76	1
8p	29	18	8	7	61.7	53.3	0.76	1
22q	30	18	9	7	62.5	56.3	0.77	1
4p	28	21	8	8	57.1	50.0	0.77	1
21q	24	23	10	7	51.1	58.8	0.78	1
17p	34	13	11	4	72.3	73.3	1.00	1
18q	39	9	13	3	81.3	81.3	1.00	1
7q	5	39	2	14	11.4	12.5	1.00	1
9p	17	24	6	9	41.5	40.0	1.00	1
12q	12	31	4	9	27.9	30.8	1.00	1
18p	39	9	12	3	81.3	80.0	1.00	1
4q	24	15	8	5	61.5	61.5	1.00	1
5p	13	32	4	12	28.9	25.0	1.00	1
7p	5	42	2	13	10.6	13.3	1.00	1
8q	15	31	4	10	32.6	28.6	1.00	1
16p	17	31	6	10	35.4	37.5	1.00	1
5q	20	16	5	5	55.6	50.0	1.00	1
16q	18	26	6	10	40.9	37.5	1.00	1
14q	32	15	12	6	68.1	66.7	1.00	1

p-values were calculated using two-sided Fisher's exact test. Multiple hypothesis was performed using Benjamini-Hochberg Procedure.
Abbreviations- del & loss, Deletion and Loss, No-Del or Loss, No Deletion or Loss, Freq, Frequency.

© 2025 Omer DM. *JAMA Network Open*.

eTable 9. Mutational signature comparison from WES data (SRC vs. PRC)								
	**Positive SRC samples (n)**	**Negative SRC samples (n)**	**SRC positive Freq (%)**	**Positive PRC samples (n)**	**Negative PRC samples (n)**	**PRC positive Freq (%)**	**p-value**	**Adjusted p-value**
Signatures of unknown aetiology	11	6	64.7	2	26	7.1	0.00	0.00
Defective DNA base excision repair due to *NTHL1* mutations	3	14	17.6	0	28	0.0	0.05	0.25
POLE	3	14	17.6	0	28	0.0	0.05	0.25
Tobacco chewing	4	13	23.5	1	27	3.6	0.06	0.25
MSI	5	12	29.4	4	24	14.3	0.27	0.80
UV	3	14	17.6	2	26	7.1	0.35	0.80
Defective homologous recombination DNA damage repair	1	16	5.9	0	28	0.0	0.38	0.80
Azathioprine treatment	1	16	5.9	0	28	0.0	0.38	0.80
Tobacco smoking	0	17	0.0	0	28	0.0	1.00	1.00
Polymerase eta somatic hypermutation activity	0	17	0.0	0	28	0.0	1.00	1.00
Damage by reactive oxygen species	0	17	0.0	0	28	0.0	1.00	1.00
Aflatoxin exposure	3	14	17.6	4	24	14.3	1.00	1.00
Haloalkane exposure	0	17	0.0	0	28	0.0	1.00	1.00
Aging	0	17	0.0	0	28	0.0	1.00	1.00
APOBEC	0	17	0.0	0	28	0.0	1.00	1.00
Sequencing artifacts	4	13	23.5	6	22	21.4	1.00	1.00
Chemotherapy	0	17	0.0	0	28	0.0	1.00	1.00

p-values were calculated using two-sided Fisher's exact test. Multiple hypothesis testing was performed using Benjamini-Hochberg Procedure.
Abbreviations- Freq, Frequency.

© 2025 Omer DM. *JAMA Network Open*.

eFigure 4. Extended results of mutational patterns using WES from SRC and PRC patients

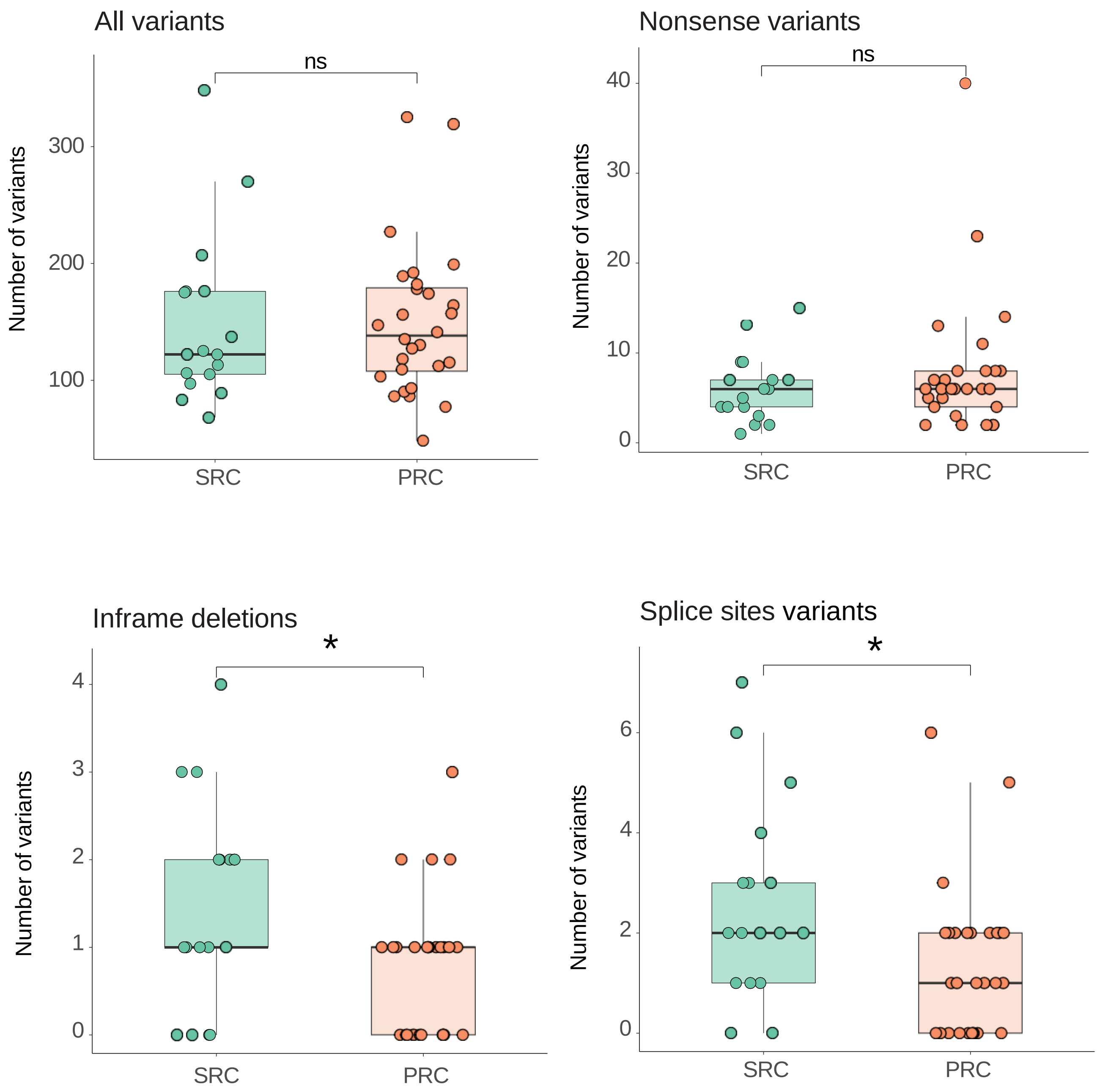

A. Number of variants identified using WES of specimens from the SRC and the PRC cohorts, broken down by variant classes. Results are shown for all types of variants, nonsense variants, inframe deletions and splice site variants. p value was calculated using the Wilcoxon test. Abbreviations- n.s: not significant, p>0.05, * p≤0.05, ** p≤0.01, *** p≤0.001, **** p≤0.0001.

© 2025 Omer DM. *JAMA Network Open*.
