# Supplementary material for: Clinical and Genomic Characterization of Secondary Rectal Cancer After Radiotherapy for Prostate Cancer
Source: JAMA Netw Open. 2025 Mar 18;8(3):e251039. doi: 10.1001/jamanetworkopen.2025.1039 (PMC11920846; doi:10.1001/jamanetworkopen.2025.1039)
Supplement: Supplement 3. — Data Sharing Statement [file jamanetwopen-e251039-s003.pdf]

Data Sharing Statement

Omer. Clinical and Genomic Characterization of Secondary Rectal Cancer After Radiotherapy for Prostate Cancer. *JAMA Netw Open*. Published March 18, 2025.
doi:10.1001/jamanetworkopen.2025.1039

Data
Data available: Yes
Data types: Deidentified participant data
How to access data: https://www.cbioportal.org/study/summary?id=rectal_radiation_msk_2024.
When available: With publication

Supporting Documents
Document types: None

Additional Information
Who can access the data: Anyone requesting the data.
Types of analyses: For any purpose.
Mechanisms of data availability: Open access through cBioPortal for Cancer Genomics: doi:10.1126/scisignal.2004088, doi:10.1158/2159-8290.CD-12-0095.
